# Dataset of Vietnamese teachers’ habits and motivation behind continuous professional development programs participation

**DOI:** 10.1016/j.dib.2020.106525

**Published:** 2020-11-14

**Authors:** Thanh-Thao Thi Phan, Ngoc-Thuy Ta, Viet-Anh Phu Duong, Anh-Duc Hoang

**Affiliations:** a Reduvation Research Group, Thanh Do University, Hanoi 100000, Viet Nam; b EdLab Asia Educational Research and Development Centre, Hanoi 100000, Viet Nam; c School of Business and Management, RMIT University Vietnam, Hanoi 100000, Viet Nam

**Keywords:** Teacher professional development, Continuous professional development, K-12 School, Vietnam, Public education, Private education

## Abstract

In every educational system, teacher development has a vital role in its sector and the health of the social, cultural, and economic sectors. For this redeeming feature, all stakeholders such as education policymakers, school superintendents, and school faculties make a big room for teachers' improvement throughout continuous professional development (CPD) provisions. However, to embark on a new educational adventure is a challenging target to meet, especially when the teacher frames their teaching and learning concept years after years. We decided to survey Vietnamese teachers' habits and motivation to trace their origin back to teachers' partaking reason in these programs. This dataset acquisition occurred from 24 Sep 2019 to 26 Mar 2020 and approached public and private schools (using traditional, bi-lingual, or international curriculum). Overall, the dataset includes 464 observations (263 Vietnamese teachers and 202 expatriate teachers) from 48 K-12 schools across Vietnam. The researchers divided this dataset into three main sections, including (i) The demographic information; (ii) Teacher's CPD habits; and (iii) Teacher's perceptions concerning Project-based and Problem-based Learning (PBL).

## Specifications Table

**Table:** 

Subject	Education, Education Management
Specific subject area	Teacher Development
Type of data	Raw data in excel file and analyzed data
How data were acquired	Data was gathered using an online survey
Data format	Raw Analyzed
Parameters for data collection	This research focuses on K-12 Vietnamese teacher's habits and motivation in CPD activities
Description of data collection	An online survey has been distributed throughout randomly selected schools (from 24 Sep 2019 to 26 Mar 2020)
Data source location	Information is collected from secondary student institutes in Vietnam (Latitude 16°0′N, Longitude 106°0′E)
Data accessibility	Repository name: Mendeley Data Data identification number: Direct URL to data: https://data.mendeley.com/datasets/s9by3yvh9t/1

## Value of the Data

• This dataset would be useful for future reference in educational policymakers and practitioners to tackle the shortage of CPD provisions as well as launch fresh initiatives for these career development activities.
• This dataset demonstrates the differences in the CPD habits of teachers from public schools and private schools.
• This presented data provided an important aspect, though often ignored when launching a CPD program - teachers’ motivation.
• The dataset will help instructional coaches and consultants improve PBL units as it mentioned teachers’ perceptions when they applied these student-centric types of teaching.

## 1 Data Description

Teacher, or broadly speaking, teaching, is always regarded as one of the most respected professions. However, a significant challenge adaptation requirement, especially the day-to-day education, is continuously changing and expected to perform sustainably [1]. That explains why the essential demand CPD requirements and these supporting CPD activities such as PBL Learning are so meaningful to today's worldwide educational landscape because teaching, CPD is getting all professionals acquaintance [2,3]. In the teaching profession, CPD acted in its capacity as an advisory competence-keeper for teachers throughout a flurry of learning-related activities [4,5]. These different activities are considered virtually indispensable for teachers due to the occupational characteristics itself, in which teachers stay abreast of any sweeping changes in learning approaches, teaching methods, or advanced technologies [6,7,8].

**Table 1:** Demographic information of respondents.

Variable	Frequency	Percent
***Country***	***464***	***100***
Vietnam	263	56.7
America	72	15.5
Australia and New Zealand	62	13.4
Europe	67	14.4
***School type***	***464***	***100***
Public	138	29.7
Private (Normal curriculum)	145	31.3
Private (Bilingual curriculum)	140	30.2
Private (International curriculum)	41	8.8
***Curriculum type***	***464***	***100***
National Curriculum	146	31.5
Extended from National	219	47.2
IB Curriculum	23	5
Cambridge	76	16.4
***Teaching grade***	***464***	***100***
Primary	161	34.7
Secondary	157	33.8
High school	146	31.5
***Gender***	***464***	***100***
Male	120	25.9
Female	344	74.1
***Years of teaching experience***	***464***	***100***
Less than 3	97	20.9
Less than 5	155	33.4
Less than 10	117	25.2
More than 10	95	20.5
***Years of teaching experience at the current school***	***464***	***100***
Less than 3	232	50
Less than 5	161	34.7
Less than 10	58	12.5
More than 10	13	2.8
***Number of countries have ever taught***	***464***	***100***
1	236	50.9
2	68	14.7
3	128	27.6
4	26	5.6
5	1	0.2
6	5	1.1
***English proficiency***	***464***	***100***
Beginning	5	1.1
B1	116	25
B2	47	10.1
C1	53	11.4
C2	14	3
Native	229	49.4
***Education degree***	***464***	***100***
BA in Edu	353	76.1
MA in Edu	86	18.5
Dr in Edu	7	1.5
BA other	14	3
MA in other	4	0.9

**Table 2:** Descriptive statistic of Continuing Professional Development Habits and Motivation of teachers (*N* = 464).

				Mean			
Variables	Range	Min	Max	*Statistic*	*Std. Error*	Std. Deviation	Variance
***13. Total hours of attended courses/workshops/seminars during the past 12 months***	80	30	110	57.99	0.73	15.71	246.83
***14. In particular, the number for each kind of attended activity...***							
14.1 Online courses (self-pace)	14	0	14	4.80	0.15	3.18	10.08
14.2 Online course (interactive)	14	0	14	2.92	0.13	2.86	8.19
14.3 Online webinar	16	0	16	2.08	0.12	2.61	6.82
14.4 Attended hours are...[Offline courses (being taught)	26	0	26	10.56	0.23	4.96	24.62
14.5 Offline workshops (interactive activities)	38	0	38	16.27	0.40	8.51	72.39
14.6 Offline seminars/conferences	39	5	44	21.38	0.33	7.07	49.91
***Total activities attended during the past 12 months***	29	0	29	15.15	0.26	5.58	31.16
***15. In particular, those activities were financed by...***							
15.1 By your own money	8	0	8	2.59	0.09	1.93	3.72
15.2 School's support	13	0	13	4.53	0.10	2.23	4.99
15.3 The activities are free	12	0	12	4.51	0.13	2.84	8.08
15.4 Government's scholarship	3	0	3	0.92	0.04	0.91	0.83
15.5 Business' scholarship/sponsorship	5	0	5	1.01	0.05	1.10	1.20
15.6 NGO' scholarship/sponsorship	4	0	4	0.80	0.04	0.90	0.81
15.7 Friend's support	4	0	4	0.80	0.04	0.95	0.91
***16. I often share the know-how back to other colleagues...***							
16.1 Right after the attendance	4	1	5	3.17	0.06	1.20	1.44
16.2 A week after the attendance	3	2	5	3.77	0.05	0.99	0.98
16.3 A month after the attendance	4	1	5	2.97	0.06	1.28	1.65
16.4 Two months after the attendance	4	1	5	2.48	0.05	1.12	1.25
***17. My motivation toward those activities***							
17. 1 To enhance my overall knowledge	4	1	5	3.46	0.05	1.14	1.30
17.2 To enhance my domain knowledge	4	1	5	3.77	0.05	0.99	0.98
17.3 To get promotion	4	1	5	2.80	0.06	1.23	1.51
17.4 To have better pedagogical skills	4	1	5	3.94	0.04	0.93	0.87
17.5 To have better technical skills	4	1	5	2.84	0.05	1.05	1.10
17.6 To fulfill the requirement of my school/school district	4	1	5	4.01	0.04	0.86	0.75
17.7 Encouraged by my colleagues	4	1	5	3.08	0.05	1.09	1.18
17.8 Encouraged by my leaders	3	1	4	2.59	0.04	0.89	0.79

**Table 3:** Descriptive statistic of Teacher's experience and perception of Project-based and Problem-based learning (*N* = 464).

					Mean			
Variables		Range	Min	Max	*Statistic*	*Std. Error*	Std. Deviation	Variance
***18. Total Hours of PBL training/learning I received***		148	2	150	29.02	0.87	18.68	348.87
***19. A driving question is a must have***		4	1	5	3.63	0.05	1.11	1.23
***20. How detail a driving question should be***		3	2	5	3.47	0.05	1.01	1.01
***21. I prefer student-centric or teacher-centric educational approaches***		9	1	10	4.80	0.08	1.80	3.24
***22. Students cannot acquire the learning outcomes without the teacher's instruction***		4	1	5	3.21	0.05	1.12	1.26
***23. The learning targets cannot be reached with driving question formed by students only***		4	1	5	3.03	0.06	1.22	1.48
***24. Challenges for teachers to form driving questions***								
24.1 Time-consuming (before the lesson)		3	2	5	4.02	0.04	0.85	0.73
24.2 Time-consuming (during the lesson)		4	1	5	2.99	0.06	1.28	1.63
24.3 Adjust the questions to fit all students		4	1	5	3.46	0.05	1.03	1.06
***25. Challenges for students to form driving questions***								
25.1 Weak decision-making skills		4	1	5	3.41	0.06	1.20	1.45
25.2 Lack of know-how about the big picture		4	1	5	3.17	0.06	1.28	1.64
25.3 Time-consuming (before the lesson)		4	1	5	2.98	0.05	1.12	1.25
25.4 Time-consuming (during the lesson)		4	1	5	3.73	0.05	1.01	1.01
25.5 Lack of proactive learning habit		4	1	5	3.25	0.05	1.06	1.11
25.6 Difficult to reach alignment		4	1	5	3.23	0.05	1.07	1.15
***26. Teachers should form a driving question***		4	1	5	3.23	0.05	1.09	1.19
***27. Students should form a driving question***		3	1	4	2.33	0.05	1.01	1.02
***28. Teachers and students should form a driving question together***		4	1	5	3.05	0.05	1.14	1.31
***29. I am willing to let students decide their learning pathway***		4	1	5	3.22	0.05	1.14	1.31
***30. I am willing to let students form driving questions by themselves***		4	1	5	2.98	0.05	1.09	1.19
***31. My lesson often starts with...***								
31.1 A driving question provided by me		3	2	5	3.91	0.04	0.93	0.87
31.2 A list of driving questions provided by me		4	1	5	2.72	0.05	1.04	1.08
31.3 A driving question provided by students		4	1	5	2.32	0.04	0.93	0.87
***32. In my class, driving questions are generated...***								
32.1 After brainstorming/discussion sessions		4	1	5	2.66	0.04	0.95	0.89
32.2 To follow previous discussions		4	1	5	3.01	0.05	1.08	1.16
***33. The student: teacher ratio in my classes***		37	8	45	26.34	0.48	10.41	108.44

**Table 4:** Relationship between CPD Habits of teachers and their experience and perception of PBL.

Variables	Total hours	Total activities	Knowledge sharing	Motivation
***18. Total hours of PBL training/learning***	.367⁎⁎	0.078	0.043	−0.047
***19. A driving question is a must have***	.360⁎⁎	.232⁎⁎	0.029	−0.040
***20. How detail a driving question should be***	−0.285⁎⁎	−0.119*	0.019	0.043
***21. I prefer student-centric or teacher-centric educational approaches***	−0.021	−0.135⁎⁎	−0.055	−0.004
***22. Students cannot acquire the learning outcomes without the teacher's instruction***	−0.235⁎⁎	−0.122⁎⁎	−0.055	0.061
***23. The learning targets cannot be reached with formed driving question from students***	−0.394⁎⁎	−0.213⁎⁎	−0.039	−0.002
***24. Challenges for teachers to form driving questions***				
24.1 Time-consuming (before the lesson)	.181⁎⁎	0.078	0.025	0.001
24.2 Time-consuming (during the lesson)	.224⁎⁎	.148⁎⁎	−0.116*	−0.028
24.3 Adjust the questions to fit all students	.245⁎⁎	−0.069	−0.100*	−0.066
***25. Challenges for students to form driving questions***				
25.1 Weak decision-making skills	−0.236⁎⁎	−0.107*	.240⁎⁎	0.067
25.2 Lack of know-how about the big picture	.308⁎⁎	−0.155⁎⁎	0.053	0.055
25.3 Time-consuming (before the lesson)	.336⁎⁎	.259⁎⁎	.176⁎⁎	0.041
25.4 Time-consuming (during the lesson)	−0.003	−0.017	−0.088	−0.032
25.5 Lack of proactive learning habit	−0.282⁎⁎	−0.076	0.037	.094*
25.6 Difficult to reach alignment	.142⁎⁎	.122⁎⁎	−0.014	0.038
***26. Teachers should form a driving question***	−0.327⁎⁎	−0.298⁎⁎	−0.198⁎⁎	−0.106*
***27. Students should form a driving question***	0.080	.162⁎⁎	0.074124	0.023
***28. Teachers and students should form a driving question***	.276⁎⁎	.249⁎⁎	.140⁎⁎	0.059
***29. I am willing to let students decide their learning pathway***	.211⁎⁎	.215⁎⁎	0.064	0.041
***30. I am willing to let students form driving questions by themselves***	.263⁎⁎	.219⁎⁎	.108*	0.028
***31. My lesson often starts with...***				
31.1 A driving question provided by me	−0.161⁎⁎	−0.111*	0.074	0.003
31.2 A list of driving questions provided by me	.200⁎⁎	−0.035	−0.063	−0.042
31.3 A driving question provided by students	.237⁎⁎	.110*	−0.094*	−0.026
***32. The driving questions are generated...***				
32.1 After brainstorming/discussion sessions	.299⁎⁎	.290⁎⁎	0.027	0.025
32.2 To follow previous discussions	.133⁎⁎	−0.017	−0.022	0.002
***33. The student/teacher ratio in my classes***	−0.571⁎⁎	−0.395⁎⁎	−0.105*	0.004

Note:

⁎ *p* < .05 (2-tailed).

⁎⁎ *p* < .01 (2-tailed).

Furthermore, the COVID-19 pandemic duration showed the importance of teachers' continuous development when all students grew accustomed to online learning platforms, and their learning habits significantly changed. Moreover, this movement simultaneously proved the need for professional development continuity, which not only stood to benefit the ICT adoption in teaching and learning practices. Therefore, this dataset concentrates on Vietnamese teacher's habits and motivation among different demographic variables.

The questionnaire includes three parts: (i) The demographic information of teacher (12 questions); (ii) Teacher's participation habits in CPD programs (5 questions); and (iii) Teacher's perception related to Project-based Learning practices (16 questions). The full survey, code, and measurement parameters for all variables can be found on Mendeley Dataset [9].

## 2 Experimental Design, Materials and Methods

Firstly, we did an outline survey from 24 Sep 2019 to 26 Mar 2020. Its questionnaire was a 5-point Likert scale (1: totally disagree; 2: somewhat disagree; 3: neither agree nor disagree; 4: somewhat agree; 5: totally agree) based on the Google Forms. After receiving all responses and clarifying the data with validated 464 observations, we exported them as Master Excels (CSV file) to import SPSS 20, particularly in the below tables.

In this dataset, we mainly clarified Vietnamese K-12 teacher's habits concerning taking part in CPD activities. Mainly, we present it by the following regression to illustrate the relationship between their perceptions toward CPD (PER) and four aspects: total participated hours (HOUR); total engaged activity they (ACT); the knowledge they gained and shared with their colleagues (KNOW); and their motivation towards CPD activities (MOTIV). The overall regression model can be formed as follow:

PER∼β0+β1*(HOUR)+β2*(ACT)+β3*(KNOW)+β4*(MOTIV)+u

In terms of teachers' perception of CPD provisions, we examined challenges for all teachers and students to form driving questions when teachers applied Project-based or Problem-based learning. The first aspect (HOUR) covered teachers' hours (offline or online) in CPD courses, seminars, and workshops. The second aspect (ACT) are activities (free or charged; school's support; government scholarship; business sponsor or teacher must pay your own money). The third aspect (KNOW) is the variables examining teachers' sharing with their fellows after accessing new information sources between a time frame of one or two weeks. The last aspect (MOTIV) concludes teachers' motivations toward CPD programs. For example, many teachers might want to develop their brands for more straightforward promotion, improve pedagogical or technical skills, adapt their leaders' requirements, or follow their colleagues’ encouragement.

## Declaration of Competing Interest

The authors declare that they have no known competing financial interests or personal relationships which have, or could be perceived to have, influenced the work reported in this article.
